# A Pooled Analysis From Phase 2b and 3 Studies in Japan of Istradefylline in Parkinson's Disease

**DOI:** 10.1002/mds.28095

**Published:** 2020-06-05

**Authors:** Nobutaka Hattori, Hiroki Kitabayashi, Tomoyuki Kanda, Takanobu Nomura, Keizo Toyama, Akihisa Mori

**Affiliations:** ^1^ Department of Neurology Juntendo University School of Medicine Tokyo Japan; ^2^ Medical Affairs Department Kyowa Kirin Co., Ltd. Tokyo Japan; ^3^ R&D Division Kyowa Kirin Co., Ltd. Tokyo Japan

**Keywords:** efficacy, istradefylline, Japan, Parkinson's disease, treatment outcome

## Abstract

**Background:**

Characterization of patient factors associated with istradefylline efficacy may facilitate personally optimized treatment.

**Objectives:**

We aimed to examine which patient factors are associated with favorable istradefylline treatment outcomes in PD patients with motor complications.

**Methods:**

We performed a pooled analysis of data from two identical phase 2b and 3 Japanese studies of istradefylline. Logistic regression models were used to assess the association of 12 patient characteristics with favorable outcomes.

**Results:**

*Off* time reduction and increased good *on* time with istradefylline provided a significantly favorable response in patients aged ≥65 years. *Off* time reduction was more favorable in patients with ≥8‐hour daily *off* time at baseline. Improvement in UPDRS Part III was favorable in patients with UPDRS Part III baseline score ≥ 20.

**Conclusions:**

Several patient factors influenced the effect of istradefylline on motor fluctuations, motor function, activities of daily living, and clinical impression. © 2020 The Authors. *Movement Disorders* published by Wiley Periodicals, Inc. on behalf of International Parkinson and Movement Disorder Society.

Parkinson's disease (PD) is a progressive neurodegenerative disorder that affects 1% of people aged >65 years.^1^ PD treatment research has been dominated by dopaminergic therapies with levodopa, which currently is the most effective symptomatic treatment for PD.^2^ However, onset of motor complications limits pharmacological interventions.^2^ Therefore, the characterization and specific needs of patients with motor subtypes and motor complication subtypes are of interest to facilitate a personalized therapeutic approach.^3^

Istradefylline (KW‐6002) is the first selective adenosine A_2A_ receptor antagonist available in Japan and the United States for treatment of the *off* time in PD patients treated with l‐dopa‐containing preparations. Istradefylline is considered a nondopaminergic symptomatic anti‐PD pharmacotherapy,^4^, ^5^ with adenosine A_2A_ receptor antagonism in the basal ganglia, but also a lack of influence on dopaminergic receptors/enzymes, and has demonstrated antiparkinsonian effects in clinical studies.^6^, ^7^ In phase 2b and 3 clinical studies in PD patients treated with l‐dopa in Japan, istradefylline elicited a reduction in *off* time as well.^8^, ^9^

We aimed to establish which patient factors are likely to influence patient outcomes after istradefylline therapy and hence its future use in personally optimized treatment.

## Patients and Methods

### Patient Population and Study Design

We performed a pooled analysis of two Japanese studies^8^, ^9^ of istradefylline as an adjunct to l‐dopa. Both studies were identically designed, 12‐week, multicenter, randomized, double‐blind, placebo‐controlled, parallel‐group studies and enrolled PD patients with motor fluctuations. Additional details are provided in the Supporting Information Methods.

### Efficacy Outcomes

The primary efficacy outcome was change in daily *off* time from baseline to 12 weeks. Other efficacy outcomes included change in *on* time without troublesome dyskinesia (“Good” *on* time),^10^ UPDRS scores, and Clinical Global Impressions‐Improvement of illness (CGI‐I) score from baseline. Treatment effect was determined as the difference in mean change during follow‐up between the placebo and istradefylline arms. Definitions of the cut‐off values for efficacy outcomes are described in the Supporting Information Methods.

### Statistical Analysis

Patient background characteristics for both the total patients and the groups stratified by treatment arms are summarized using descriptive statistics. We conducted the following analyses only for patients without any missing data. To explore demographic factors associated with favorable outcomes following treatment with istradefylline, a logistic regression model was applied to estimate the odds ratio (OR) and 95% confidence interval after controlling for 12 baseline factors in three steps (described in the Supporting Information Methods). Prediction models were constructed with five outcomes (details are described in the Results.) with reference to the results of the multivariable logistic regression model as an exploratory analysis. Model performance was evaluated by the area under the curve from receiver operating characteristics curves. Presence of statistical significance and effect modification were considered with a two‐tailed *P* value <0.05 and an interaction term <0.10, respectively.^11^ All analyses were performed using SAS software (version 9.2 or 9.3; SAS Institute, Inc., Cary, NC).

## Results

### Patients

The patient disposition for the studies is presented in Supporting Information Figure S1. The total pooled full analysis set population was 723 (placebo, n = 241; istradefylline, 20 mg/d n = 235 and 40 mg/d n = 247). Most demographic and baseline characteristics were comparable between the treatment arms within the two studies (Supporting Information Table S1), except for concomitant anti‐PD drugs used at baseline, because zonisamide was not available during the phase 2b study.

### Efficacy

**FIG. 1.**
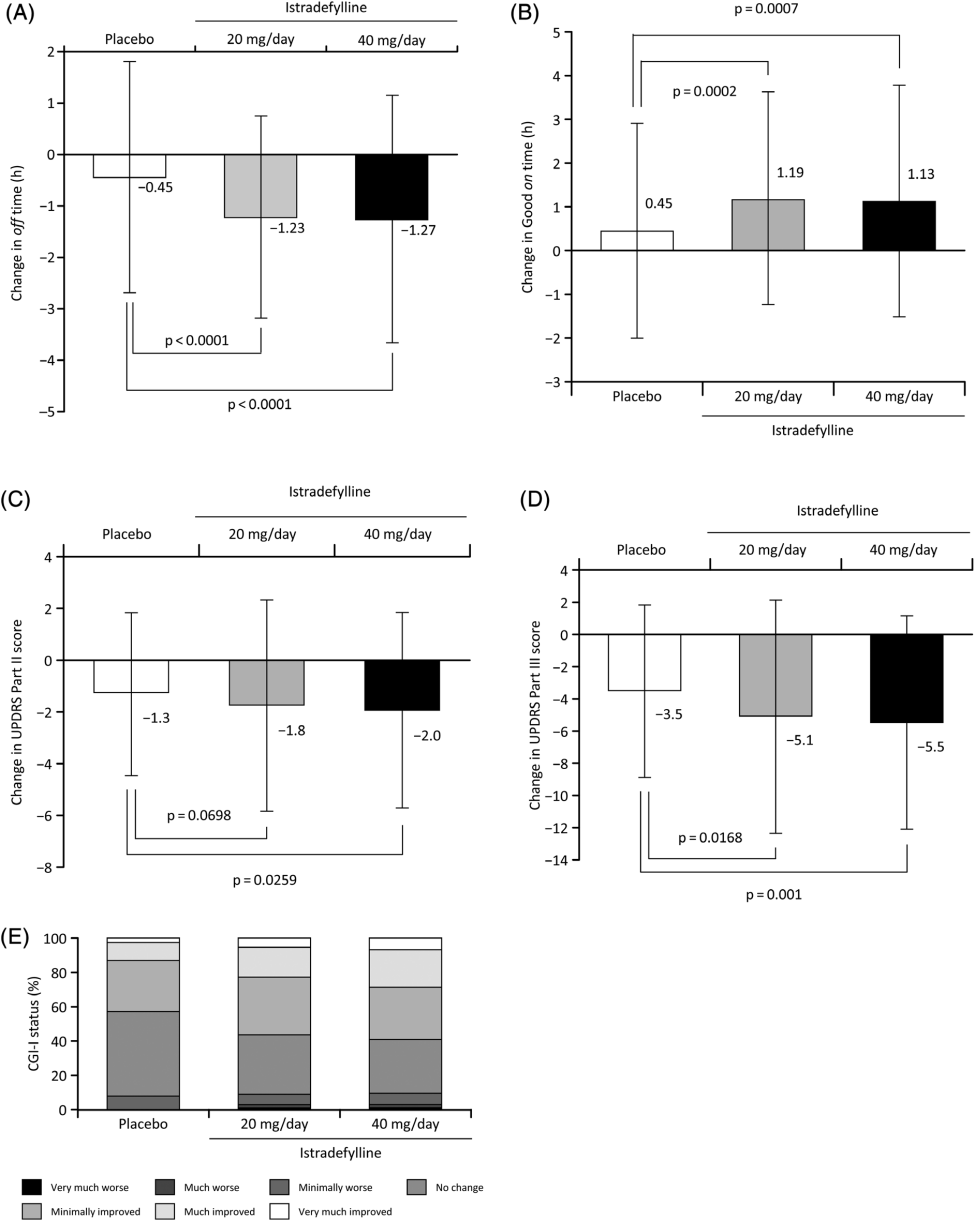
Changes from baseline at week 12 in mean daily *off* time (A), daily *on* time without troublesome dyskinesia (Good *on* time) (B), UPDRS Part II (*off*; C), UPDRS Part III (D), and CGI‐I (E) in each treatment group. Data are presented as means and standard deviations for each treatment group in panels (A), (B), (C), and (D), with *P* values for each comparison.

The overall efficacy of istradefylline is described in Figure 1, Supporting Information Figure S2, and Supporting Information Table S2. Data distributions for each treatment group were comparable among groups. Compared with placebo, both istradefylline doses were associated with significant reductions in daily *off* time as well as a significant increase in Good *on* time. Significant improvements in UPDRS Part III score (*on*) were also observed for both doses compared with placebo.

### Association Between Efficacy and Patient Demographic Factors by Multivariate Logistic Regression Analysis

We analyzed the association between the five outcomes (1, *off* time reduction; 2, increase in Good *on* time; 3, improvement in UPDRS Part II score [*off* state]; 4, improvement in UPDRS Part III score; and 5, CGI‐I score) and 12 interaction factors (1, age; 2, sex; 3, presence or absence of dyskinesia at baseline; 4, mean daily *off* time; 5, total UPDRS Part III score; 6, *on* state on Modified H & Y [mH&Y] scale; 7, *off* state on mH&Y scale; 8, pattern of concomitant anti‐PD drugs; 9, duration of PD; 10, duration of motor complication; 11, l‐dopa dosage; and 12, l‐dopa‐equivalent dose). The results are presented in Table 1.

**TABLE 1.** Correlation between effectiveness and patient demographic factors by multivariate logistic regression analysis

		*Off* Time		*P* for Heterogeneity		*On* Time Without Troublesome Dyskinesia		*P* for Heterogeneity		UPDRS Part II Score (*off*)		*P* for Heterogeneity		UPDRS Part III Score		*P* for Heterogeneity		CGI‐I		*P* for Heterogeneity
Characteristic		20 mg/d	40 mg/d			20 mg/d	40 mg/d			20 mg/d	40 mg/d			20 mg/d	40 mg/d			20 mg/d	40 mg/d	
																				
Age, y				n.s.				<0.10				n.s.				n.s.				n.s.
	<65	1.00 (ref)				1.00 (ref)	1.00 (ref)			1.00 (ref)				1.00 (ref)				1.00 (ref)		
	≥65	***2.65*** [1.65–4.28]				1.47 [0.76–2.85]	***2.88*** [1.41–5.89]			0.90 [0.56–1.46]				0.65 [0.40–1.06]				1.17 [0.74–1.85]		
																				
Sex				n.s.				n.s.				n.s.				n.s.				n.s.
	Male	1.00 (ref)				1.00 (ref)				1.00 (ref)				1.00 (ref)				1.00 (ref)		
	Female	0.86 [0.55–1.36]				0.85 [0.54–1.35]				***1.71*** [1.06–2.74]				***1.65*** [1.01–2.69]				1.56 [1.00–2.44]		
																				
Combinations of concomitant drug				<0.10				n.s.				n.s.				n.s.				n.s.
	l‐dopa, L‐dopa + DA	1.00 (ref)	1.00 (ref)			1.00 (ref)				1.00 (ref)				1.00 (ref)				1.00 (ref)		
	L‐dopa + DA + SEL/ENT/ZNS	2.18 [0.97–4.87]	0.63 [0.28–1.45]			1.07 [0.62–1.84]				1.34 [0.75–2.37]				1.20 [0.67–2.17]				0.94 [0.54–1.61]		
	L‐dopa + DA + SEL/ENT/ZNS + AMA	1.11 [0.43–3.03]	0.85 [0.29–2.46]			0.85 [0.43–1.70]				***2.28*** [1.12–4.64]				1.57 [0.75–3.28]				1.03 [0.52–2.04]		
																				
Duration of PD, y				n.s.				n.s.				n.s.				n.s.				n.s.
	<5	1.00 (ref)				1.00 (ref)				1.00 (ref)				1.00 (ref)				1.00 (ref)		
	5 to <10	1.32 [0.78–2.25]				1.43 [0.84–2.43]				1.41 [0.82–2.41]				1.04 [0.60–1.81]				1.23 [0.73–2.06]		
	≥10	1.41 [0.72–2.75]				1.78 [0.92–3.48]				1.07 [0.53–2.15]				0.82 [0.41–1.63]				1.21 [0.63–2.33]		
																				
Duration of motor complications, y				n.s.				n.s.				n.s.				n.s.				n.s.
	<3	1.00 (ref)				1.00 (ref)				1.00 (ref)				1.00 (ref)				1.00 (ref)		
	≥3	0.98 [0.60–1.59]				1.01 [0.63–1.64]				0.93 [0.57–1.53]				1.07 [0.65–1.78]				1.08 [0.67–1.73]		
L‐dopa dose, mg/d				n.s.				n.s.				n.s.				<0.10				n.s.
	<400	1.00 (ref)				1.00 (ref)				1.00 (ref)				1.00 (ref)	1.00 (ref)			1.00 (ref)		
	≥400	0.80 [0.48–1.35]				0.80 [0.48–1.34]				0.98 [0.57–1.67]				1.31 [0.55–3.14]	0.72 [0.36–1.46]			0.71 [0.42–1.19]		
																				
L‐dopa‐equivalent dose, mg/d				n.s.				n.s.				n.s.				<0.10				n.s.
	<700	1.00 (ref)				1.00 (ref)				1.00 (ref)				1.00 (ref)	1.00 (ref)			1.00 (ref)		
	≥700	0.96 [0.56–1.67]				0.88 [0.51–1.50]				0.81 [0.46–1.42]				1.04 [0.43–2.50]	0.63 [0.29–1.37]			1.22 [0.71–2.09]		
																				
Dyskinesia on baseline				n.s.				n.s.				n.s.				<0.10				<0.10
	Presence	1.00 (ref)				1.00 (ref)				1.00 (ref)				1.00 (ref)	1.00 (ref)			1.00 (ref)	1.00 (ref)	
	Absence	0.74 [0.46–1.20]				0.81 [0.50–1.32]				***2.27*** [1.37–3.85]				0.87 [0.41–1.84]	1.82 [0.88–3.70]			0.79 [0.40–1.59]	***2.27*** [1.10–4.76]	
																				
Mean daily *off* time, h				<0.10				<0.10				n.s.				n.s.				n.s.
	<4	1.00 (ref)	1.00 (ref)			1.00 (ref)	1.00 (ref)			1.00 (ref)				1.00 (ref)				1.00 (ref)		
	4 to <8	1.27 [0.53–3.05]	1.58 [0.66–3.77]			1.09 [0.47–2.56]	0.58 [0.25–1.34]			1.00 [0.55–1.83]				0.85 [0.47–1.55]				1.09 [0.61–1.94]		
	≥8	1.57 [0.63–3.92]	***6.68*** [2.41–18.5]			1.19 [0.49–2.91]	2.38 [0.91–6.21]			0.98 [0.51–1.89]				0.53 [0.27–1.03]				0.85 [0.45–1.60]		
																				
UPDRS Part III				n.s.				n.s.				n.s.				n.s.				n.s.
	<20	1.00 (ref)				1.00 (ref)				1.00 (ref)				1.00 (ref)				1.00 (ref)		
	≥20	0.88 [0.56–1.36]				0.97 [0.63–1.50]				0.79 [0.50–1.25]				***2.79*** [1.75–4.46]				1.49 [0.97–2.30]		
																				
mH&Y scale (*on*)				n.s.				n.s.				n.s.				<0.10				n.s.
	<3	1.00 (ref)				1.00 (ref)				1.00 (ref)				1.00 (ref)	1.00 (ref)			1.00 (ref)		
	≥3	1.10 [0.65–1.84]				0.96 [0.57–1.61]				0.72 [0.42–1.23]				0.54 [0.24–1.21]	1.21 [0.57–2.58]			0.73 [0.44–1.21]		
																				
mH&Y (*off*)				n.s.				n.s.				<0.10				n.s.				n.s.
	<3	1.00 (ref)				1.00 (ref)				1.00 (ref)	1.00 (ref)			1.00 (ref)				1.00 (ref)		
	≥3	0.63 [0.35–1.15]				0.60 [0.33–1.08]				0.99 [0.41–2.39]	***3.50*** [1.36–9.00]			1.81 [0.95–3.46]				***1.89*** [1.07–3.36]		

Data are presented as odds ratio and 95% confident intervals, which were obtained by multivariate logistic regression analyses controlling for 12 baseline factors. Type III *P* values were calculated from multivariate logistic regression analyses controlling for the same 12 baseline covariates plus istradefylline dose (20 or 40 mg/d) and corresponding interaction terms.

ref, reference value; n.s., not significant; DA, dopamine agonist; SEL, selegiline; ENT, entacapone; AMA, amantadine; ZNS, zonisamide. Cut‐off values for the treatment effect used to determine the effectiveness were based on clinically meaningful changes, a difference from placebo (except CGI‐I), as follows: a reduction in *off* time of ≥1 hour, an increase in Good *on* time of ≥1 hour, a reduction of UPDRS Part III score of ≥3 points, a reduction of UPDRS Part II score of ≥1 point, CGI‐I score “minimally improved” or better (see the Supporting Information Method).

#### Off Time Reduction

The reduction in *off* time as the primary efficacy outcome was associated with istradefylline treatment, and the effectiveness was significantly greater in patients aged ≥65 years (OR, 2.65). Patients with higher baseline *off* time showed a significantly greater reduction of *off* time with the 40‐ versus 20‐mg/d dose of istradefylline or a lower baseline *off* time. The effect of istradefylline at 40 mg/d on *off* time reduction was most favorably observed in patients with ≥8 hours of daily *off* time at baseline (OR, 6.68).

#### Other Efficacy Endpoints

The influence of istradefylline 40 mg/d on the increase in Good *on* time was significantly greater in patients aged ≥65 years (OR, 2.88). Female sex (OR, 1.65) and higher baseline of UPDRS Part III score (OR, 2.79) were identified as factors associated with favorable improvement in UPDRS Part III score following istradefylline treatment. Female sex (OR, 1.71), absence of baseline dyskinesia (OR, 2.27), treatment with l‐dopa + anti‐PD medications including amantadine (OR, 2.28), and baseline mH&Y stage (*off* state) score ≥ 3 (OR, 3.50) were identified as factors associated with favorable improvement UPDRS Part II score (*off* state) following treatment with istradefylline. An mH&Y stage (*off* state) ≥3 (OR, 1.89) and absence of baseline dyskinesia (OR, 2.27) were associated with an improvement in CGI‐I score following treatment with istradefylline (dosages of istradefylline are described in Table 1).

## Discussion

Our analysis revealed that the effects of istradefylline on *off* time were more favorable in patients aged ≥65 years. Istradefylline elicited significant increases in Good *on* time. Similar to the results for *off* time, age ≥ 65 years was significantly associated with a more favorable trend in Good *on* time. This age group accounted for 57% of patients in our analysis. However, although the number of patients aged ≥65 versus <65 years was balanced, this does not reflect the real‐world setting, in which the overwhelming majority of PD patients are elderly.^1^ Thus, istradefylline may be useful as adjunct therapy in terms of the *off* time reduction/Good *on* time increase for the majority of PD patients.

Patients with longer *off* time at baseline were more likely to have favorable outcomes, but the reason for this is not clearly understood given that there were no significant differences in baseline l‐dopa dose or l‐dopa‐equivalent dose in this subpopulation; therefore, these patients may have not received maximal treatment benefits owing to dose limitations related to dopaminergic side effects. As an important secondary outcome, UPDRS Part III also indicated that baseline score ≥ 20 was associated with more favorable outcomes. This suggests that the effects of istradefylline are more easily detected in terms of wearing‐off, but also motor dysfunction in a wide therapeutic window (i.e., patients with longer baseline *off* time or higher baseline UPDRS Part III score).

It has been suggested that adenosine A_2A_ receptors abrogate the dopamine D2 receptor‐mediated inhibitory influence on the indirect pathway.^12^ When A_2A_ receptors are blocked, the normal function of D2 receptors on the pathway is restored. Thus, the efficacy of A_2A_ receptor antagonists may depend on individual patient variability in the extent to which excitability of indirect pathway can be regulated by D2 receptors. This proposed dopamine D2 receptor‐mediated “therapeutic window” may be supported by a monkey study, in which combination treatment of istradefylline with threshold dopaminergic therapy elicited remarkable and significant improvement in efficacy in an MPTP model,^13^ although this needs clarification in clinical studies.

Sex was an influential factor, given that more favorable improvements in UPDRS Part III and Part II (*off* state) were observed in female patients. Although male sex is a risk factor for PD,^14^ no reports have found sex differences for the pharmacology or toxicology of istradefylline. We found a difference in mean body weight between sexes (female, 49.40 kg; male, 62.56 kg) in the istradefylline‐treated arms. Thus, istradefylline exposure in the body (mg/kg) may be higher in female than male patients; hence, being female could be more favorable for improvement in UPDRS Parts II and III, although this remains to be investigated with a larger sample size.

The multivariate logistic regression analysis indicated that baseline mH&Y (*off* state) ≥3 and lack of dyskinesia at baseline were associated with more favorable CGI‐I outcomes. Dyskinesia at baseline may influence the effect of istradefylline on improvements in CGI‐I, given that dyskinetic movement affects the impression of drug efficacy.^15^

This study identified some demographic factors associated with favorable istradefylline treatment outcomes. These results suggest the potential for personal optimization of therapy using the same drug in different patients who desire different clinical efficacy outcomes: (1) Patients aged ≥65 years can expect more favorable effects on motor fluctuations without troublesome dyskinesia; (2) patients with a wider therapeutic window at baseline in daily *off* time and UPDRS Part III score can expect more favorable effects on *off* time reduction and improvement of UPDRS Part III score; (3) patients with a higher mH&Y stage can expect more favorable effects in UPDRS Part II score and CGI‐I improvement; and (4) patients without pre‐existing dyskinesia can expect more favorable effects with istradefylline in terms of CGI‐I and UPDRS Part II *off* state (activities of daily living).

Limitations of this pooled analysis include the enrolment of only Japanese patients and the relatively short duration of each study.

In conclusion, istradefylline exerts an effect on the wearing‐off phenomenon in Japanese PD patients treated with l‐dopa. This analysis also suggested favorable pairings between patient factors and clinical endpoints, providing useful information for patient selection and prognostic indicators for particular characteristics. Our findings provide the first insight into adenosine A_2A_ receptor antagonist‐based personalized PD therapy, tailored according to its desired outcomes in individual clinical subtypes, without considering a patient's genetic background.

## Author Roles

(1) Research Project: A. Conception and Design, B. Acquisition of Data, C. Analysis and Interpretation of Data; (2) Manuscript: A. Writing of the First Draft, B. Review and Critique; (3) Other: A. Study Conduct, B, Approved the Final Version and Agreed to Be Accountable for All Aspects of the Work.

N.H.: 1C, 2A, 2B, 3B

H.K.: 1B, 1C, 2A, 2B, 3B

T.N.: 1B, 1C, 2A, 2B, 3B

T.K.: 1A, 1C, 2A, 2B, 3A, 3B

A.M.: 1A, 1C, 2A, 2B, 3A, 3B

K.T.: 1B, 2B, 3B

## Financial Disclosures

N.H. has received honoraria for manuscript writing and advisory board fees from Kyowa Kirin Co., Ltd; has received grants from the Ministry of Education, Culture, Sports, Science, and Technology Japan, the Japan Agency for Medical Research and Development (AMED), the Japan Society for the Promotion of Science (JSPS), and Ono Pharmaceutical Co., Ltd.; and personal fees/other support from the International Parkinson and Movement Disorder Society, Sumitomo Dainippon Pharma Co., Ltd., Otsuka Pharmaceutical Co., Ltd., Takeda Pharmaceutical Co., Ltd, Kyowa Kirin Co., Ltd., GSK KK, Nippon Boehringer Ingelheim, Co., Ltd., FP Pharmaceutical Corporation, Eisai Co., Ltd., Kissei Pharmaceutical Company, Nihon Medi‐physics Co., Ltd., Novartis Pharma KK, Biogen Idec Japan Ltd., AbbVie, Acorda Therapeutics, Inc., Medtronic, Inc., Astellas Pharma, Inc., IBM Japan, Boston Scientific Japan, Ono Pharmaceutical Co., Ltd., Hydrogen Health Medical Lab Co., ABIST Co., Ltd., Melodian Co., Ltd., Daiwa Co., Ltd., Bayer Yakuhin, Ltd., Nihon Pharmaceutical Co., Ltd., Asahi Kasei Medical Co., Ltd., MiZ Co., Ltd., Mitsubishi Tanabe Pharma Corporation, Daiichi Sankyo Co., Ltd., OHARA Pharmaceutical Co., Ltd., Meiji Seika Pharma, Sanofi KK, Pfizer Japan, Inc., Alexion Pharmaceuticals, Mylan NV, MSD KK, Lund Beck Japan, and Hisamitsu Pharmaceutical Co., Inc., outside the submitted work. H.K. is an employee of Kyowa Kirin Co., Ltd. and has a patent pending for an antiparkinsonian agent. T.N. is an employee of Kyowa Kirin Co., Ltd. and has a patent pending for an antiparkinsonian agent. T.K. is an employee of Kyowa Kirin Co., Ltd. and has a patent pending for an antiparkinsonian agent. K.T. is an employee of Kyowa Kirin Co., Ltd. A.M. is an employee of Kyowa Kirin Co., Ltd. and has a patent pending for an antiparkinsonian agent.

## Supporting information


**Appendix** **S1**: Supporting nformation

**Supplementary Figure 1** Patient disposition
**Supplementary Figure 2.** Histogram showing the change from baseline at week 12 in daily OFF time in each treatment group
Data are presented as means and standard deviations for each treatment group with p‐values for istradefylline versus placebo.
