# Supplementary material for: A Pooled Analysis From Phase 2b and 3 Studies in Japan of Istradefylline in Parkinson's Disease
Source: Mov Disord. 2020 Jun 5;35(8):1481–7. doi: 10.1002/mds.28095 (PMC7496465; doi:10.1002/mds.28095)
Supplement: Supplementary file 1 — Appendix S1: Supporting nformation [file MDS-35-1481-s001.docx]

**Supplementary materials**

**A Pooled Analysis from Phase 2b and 3 Studies in Japan of Istradefylline in Parkinson’s Disease**

Hattori N *et al.*

**Supplementary Methods**

***Patient population and study design***

Key inclusion criteria included age ≥20 years, stage 2–4 on the Modified Hoehn and Yahr (mH&Y) scale (OFF state), stable treatment regimen of antiparkinsonian drugs, and motor fluctuations with ≥2 hours/day OFF time. Key exclusion criteria included history of neurosurgical operation for Parkinson’s disease (PD), dementia, and pregnancy. In both studies, eligible patients were randomized to one of three arms: istradefylline 20 mg/day, istradefylline 40 mg/day, or placebo for 12 weeks. The patients attended site visits at weeks 2, 4, 8, and 12 after randomization, and completed 24-hr patient diaries covering the 7-day period prior to each visit. The full analysis set included patients who received at least one dose of study drug and returned at least one set of diaries.

***Definition of cut-off value for efficacy outcomes***

Cut-off values for the treatment effect used to determine the effectiveness of istradefylline were based on clinically meaningful changes and included a reduction in OFF time of ≥1 hour, an increase in Good ON time of ≥1 hour , and a reduction of Unified Parkinson’s Disease Rating Scale (UPDRS) Part III score of ≥3 points. The data distributions of each treatment group were comparable and placebo-subtracted.^1^ With respect to the UPDRS Part II score (OFF state), the effectiveness of istradefylline was determined via receiver operating characteristic curve between Clinical Global Impressions-Improvement of illness (CGI-I) at 12 weeks and UPDRS Part II change from baseline, and defined as a reduction of ≥1 point. Furthermore, change in CGI-I score was evaluated as “very much improved”, “much improved”, or “minimally improved”.

### **Statistical analysis**

Outcomes included OFF time, ON time without troublesome dyskinesia, UPDRS Part III score (ON state), CGI-I score, and UPDRS Part II score (OFF state). Covariates considered in the logistic regression models were age, sex, presence or absence of dyskinesia experienced at baseline (based on self-reported symptoms in 24-hr patient diary), mean daily OFF time, total UPDRS Part III score, mH&Y scale (ON/OFF), pattern of concomitant anti-PD drugs, duration of PD, duration of motor complications, levodopa dose, and levodopa-equivalent dose.

The three steps to evaluate which demographic and baseline factors were associated with the outcomes were as follows: first, we evaluated the significance of the effect modification (for heterogeneity) of each variable by two doses (20 mg or 40 mg) referring to type III p-value by multivariate logistic regression analysis, which included 12 baseline covariates with corresponding interaction terms. Second, we estimated a dose-specific odds ratio (OR) by performing dose-stratified logistic regression analysis if the variable exerted a statistically significant modification effect. Finally, we estimated an OR by logistic regression analysis if the variable did not exert an effect modification.

**Reference**

1. Hauser RA, Auinger P. Determination of Minimal Clinically Important Change in Early and Advanced Parkinson’s Disease. Movement Disorders 2011; 26: 813-818.

**Supplementary Figure 1.** Patient disposition

**Supplementary Figure 2.** Histogram showing the change from baseline at week 12 in daily OFF time in each treatment group

Data are presented as means and standard deviations for each treatment group with p-values for istradefylline versus placebo.

**Supplementary Table 1.** Demographic and baseline characteristics

**Supplementary Table 2.** Change from baseline to week 12 for each endpoint and CGI-I at week 12

# Supplementary Table 1. Demographic and baseline characteristics

|  |  |  | Istradefylline | | |
| --- | --- | --- | --- | --- | --- |
|  |  | Placebo | 20 mg/day | 40 mg/day | Total |
| Characteristic | | N=241 | N=235 | N=247 | N=482 |
|  |  |  |  |  |  |
| Age, y |  | 65.4±8.1 | 65.6±8.0 | 64.7±8.8 | 65.1±8.4 |
|  |  |  |  |  |  |
| Sex |  |  |  |  |  |
|  | Male | 103 (42.7) | 90 (38.3) | 119 (48.2) | 209 (43.4) |
|  | Female | 138 (57.3) | 145 (61.7) | 128 (51.8) | 273 (56.6) |
|  |  |  |  |  |  |
| Concomitant drug | |  |  |  |  |
|  | DA | 217 (90.0) | 213 (90.6) | 217 (87.9) | 430 (89.2) |
|  | Anticholinergic drug | 43 (17.8) | 30 (12.8) | 42 (17.0) | 72 (14.9) |
|  | SEL | 119 (49.4) | 109 (46.4) | 142 (57.5) | 251 (52.1) |
|  | ENT | 67 (27.8) | 85 (36.2) | 84 (34.0) | 169 (35.1) |
|  | AMA | 94 (39.0) | 84 (35.7) | 83 (33.6) | 167 (34.6) |
|  | ZNS | 17 (7.1) | 13 (5.5) | 20 (8.1) | 33 (6.8) |
|  |  |  |  |  |  |
| Combinations of concomitant drug | |  |  |  |  |
|  | L-DOPA, L-DOPA+DA | 51 (26.0) | 44 (23.2) | 50 (24.6) | 94 (23.9) |
|  | L-DOPA+DA+SEL/ENT/ZNS | 87 (44.4) | 97 (51.1) | 98 (48.3) | 195 (49.6) |
|  | L-DOPA+DA+SEL/ENT/ZNS+AMA | 58 (29.6) | 49 (25.8) | 55 (27.1) | 104 (26.5) |
|  |  |  |  |  |  |
| Duration of PD, y | | 8.161±4.633 | 7.661±4.150 | 7.911±4.299 | 7.789±4.225 |
|  |  |  |  |  |  |
| Duration of motor complications, y | | 3.468±3.249 | 3.175±2.629 | 3.192±2.942 | 3.184±2.791 |
|  |  |  |  |  |  |
| L-DOPA dose, mg | | 425.8±144.5 | 419.1±137.3 | 417.9±145.9 | 418.5±141.6 |
|  |  |  |  |  |  |
| L-DOPA-equivalent dose, mg | | 772.6±278.3 | 740.8±250.5 | 731.3±268.6 | 735.9±259.7 |
|  |  |  |  |  |  |
| Mean daily OFF time, h | | 6.37±2.59 | 6.67±2.79 | 6.26±2.48 | 6.46±2.64 |
|  |  |  |  |  |  |
| Mean daily percentage of OFF time, percent | | 39.20±15.44 | 40.82±16.13 | 38.36±15.18 | 39.56±15.68 |
|  |  |  |  |  |  |
| Mean daily ON time, h | |  |  |  |  |
|  | Without dyskinesia | 8.07±3.20 | 7.86±3.42 | 8.26±3.53 | 8.07±3.48 |
|  | With dyskinesia | 1.77±2.89 | 1.71±2.97 | 1.89±3.26 | 1.80±3.12 |
|  | With non-troublesome dyskinesia | 1.22±2.20 | 1.13±2.02 | 1.23±2.28 | 1.18±2.15 |
|  | With troublesome dyskinesia | 0.54±1.30 | 0.58±1.48 | 0.66±1.52 | 0.620±1.50 |
|  | Without troublesome dyskinesia | 9.28±2.63 | 9.00±2.80 | 9.49±2.81 | 9.25±2.81 |
|  |  |  |  |  |  |
| Mean daily percentage of ON time, percent | |  |  |  |  |
|  | Without dyskinesia | 49.82±19.29 | 48.61±21.17 | 50.32±20.88 | 49.49±21.02 |
|  | With dyskinesia | 10.99±17.93 | 10.58±18.35 | 11.33±19.28 | 10.97±18.82 |
|  | With non-troublesome dyskinesia | 7.62±13.72 | 6.92±12.22 | 7.41±13.49 | 7.17±12.88 |
|  | With troublesome dyskinesia | 3.37±8.06 | 3.66±9.62 | 3.93±9.01 | 3.80±9.30 |
|  | Without troublesome dyskinesia | 57.45±15.72 | 55.53±17.09 | 57.72±15.86 | 56.66±16.49 |
|  |  |  |  |  |  |
| UPDRS Part I | | 1.0±1.4 | 1.1±1.3 | 1.1±1.4 | 1.1±1.3 |
|  |  |  |  |  |  |
| UPDRS Part II | |  |  |  |  |
|  | ON | 6.1±5.2 | 5.5±4.9 | 5.7±5.0 | 5.6±4.9 |
|  | OFF | 15.2±7.4 | 15.3±7.3 | 15.6±7.7 | 15.5±7.5 |
|  |  |  |  |  |  |
| UPDRS Part III (ON) | | 21.1±10.5 | 21.2±10.7 | 20.9±11.0 | 21.0±10.8 |
|  |  |  |  |  |  |
| UPDRS Part IV | | 4.9±2.1 | 5.0±2.0 | 5.1±2.4 | 5.1±2.2 |
|  |  |  |  |  |  |
| UPDRS Parts I–III (ON) | | 28.3±14.5 | 27.8±14.1 | 27.7±14.7 | 27.7±14.4 |
|  |  |  |  |  |  |
| Modified Hoehn & Yahr scale (ON) | |  |  |  |  |
|  | 0 | 1 (0.4) | 2 (0.9) | 2 (0.8) | 4 (0.8) |
|  | 1 | 15 (6.3) | 9 (3.9) | 11 (4.5) | 20 (4.2) |
|  | 1.5 | 6 (2.5) | 6 (2.6) | 12 (4.9) | 18 (3.8) |
|  | 2 | 87 (36.6) | 85 (36.5) | 90 (36.6) | 175 (36.5) |
|  | 2.5 | 61 (25.6) | 60 (25.8) | 63 (25.6) | 123 (25.7) |
|  | 3 | 60 (25.2) | 61 (26.2) | 61 (24.8) | 122 (25.5) |
|  | 4 | 8 (3.4) | 10 (4.3) | 7 (2.8) | 17 (3.5) |
|  | 5 | 0 (0.0) | 0 (0.0) | 0 (0.0) | 0 (0.0) |
|  |  |  |  |  |  |
| Modified Hoehn & Yahr scale (OFF) | |  |  |  |  |
|  | 0 | 0 (0.0) | 0 (0.0) | 0 (0.0) | 0 (0.0) |
|  | 1 | 0 (0.0) | 0 (0.0) | 0 (0.0) | 0 (0.0) |
|  | 1.5 | 0 (0.0) | 0 (0.0) | 0 (0.0) | 0 (0.0) |
|  | 2 | 13 (5.5) | 14 (6.0) | 17 (6.9) | 31 (6.5) |
|  | 2.5 | 31 (13.0) | 27 (11.6) | 30 (12.2) | 57 (11.9) |
|  | 3 | 113 (47.5) | 100 (42.9) | 101 (41.1) | 201 (42.0) |
|  | 4 | 81 (34.0) | 92 (39.5) | 98 (39.8) | 190 (39.7) |
|  | 5 | 0 (0.0) | 0 (0.0) | 0 (0.0) | 0 (0.0) |

Data presented as mean±standard deviation or number of patients (%).

DA=dopamine agonist; SEL=selegiline; ENT=entacapone; AMA=amantadine; ZNS=zonisamide; L-DOPA=levodopa; PD=Parkinson’s disease; UPDRS=Unified Parkinson’s Disease Rating Scale.

# Supplementary Table 2. Change from baseline to week 12 for each endpoint and CGI-I at week 12

|  |  |  | Istradefylline | |
| --- | --- | --- | --- | --- |
|  |  | Placebo | 20 mg/day | 40 mg/day |
| Characteristic | | N=241 | N=235 | N=247 |
| Mean daily OFF time, hours | | −0.45±2.25 | −1.23±1.97  (p<0.0001) | −1.27±2.42  (p<0.0001) |
|  |  |  |  |  |
| Mean daily percentage of OFF time, percent | | −2.75±13.88 | −7.62±12.62  (p<0.0001) | −7.76±15.02  (p<0.0001) |
|  |  |  |  |  |
| Mean daily ON time, hours | |  |  |  |
|  | Without dyskinesia | 0.41±2.58 | 1.03±2.20  (p=0.0020) | 0.99±2.86  (p=0.0053) |
|  | With dyskinesia | −0.05±1.70 | 0.19±1.53  (p=0.9926) | 0.20±1.93  (p=0.9990) |
|  | With non-troublesome dyskinesia | 0.03±1.45 | 0.16±1.56  (p=0.0287) | 0.15±1.36  (p=0.0496) |
|  | With troublesome dyskinesia | −0.07±0.84 | 0.03±1.33  (p=0.9231) | 0.06±1.13  (p=0.9868) |
|  | Without troublesome dyskinesia | 0.45±2.46 | 1.19±2.44  (p=0.0002) | 1.13±2.65  (p=0.0007) |
|  |  |  |  |  |
| Mean daily percentage of ON time, percent | |  |  |  |
|  | Without dyskinesia | 2.90±15.85 | 6.28±13.68  (p=0.0050) | 6.46±17.10  (p=0.0085) |
|  | With dyskinesia | −0.16±10.48 | 1.32±9.36  (p=0.9817) | 1.28±11.40  (p=0.9939) |
|  | With non-troublesome dyskinesia | 0.23±8.91 | 1.17±9.54  (p=0.0442 ) | 0.92±8.26  (p=0.0910) |
|  | With troublesome dyskinesia | −0.39±5.29 | 0.15±8.12  (p=0.8890) | 0.36±6.44  (p=0.9832) |
|  | Without troublesome dyskinesia | 3.13±14.97 | 7.45±14.95  (p=0.0005) | 7.39±15.73  (p=0.0013) |
|  |  |  |  |  |
| UPDRS Part I | | −0.1±0.7 | −0.2±0.9  (p=0.2729) | −0.1±1.0  (p=0.4129) |
|  |  |  |  |  |
| UPDRS Part II | |  |  |  |
|  | ON | −0.6±1.7 | −0.5±2.3  (p=0.5691) | −0.7±2.2  (p=0.3266) |
|  | OFF | −1.3±3.2 | −1.8±4.1  (p=0.0698) | −2.0±3.8  (p=0.0259) |
|  |  |  |  |  |
| UPDRS Part III (ON) | | −3.5±5.3 | −5.1±7.2  (p=0.0168) | −5.5±6.6  (p=0.0010) |
|  |  |  |  |  |
| UPDRS Part IV | | −0.2±1.3 | −0.1±1.6  (p=0.4071) | −0.5±1.6  (p=0.0110) |
|  |  |  |  |  |
| UPDRS Parts I–III (ON) | | −4.3±6.2 | −5.8±8.6  (p=0.0494) | −6.3±8.1  (p=0.0072) |
|  |  |  |  |  |
| CGI-I | |  |  |  |
|  | Very much improved | 3 (1.3) | 6 (2.6) | 11 (4.5) |
|  | Much improved | 27 (11.3) | 43 (18.3) | 53 (21.5) |
|  | Minimally improved | 70 (29.2) | 85 (36.2) | 80 (32.5) |
|  | No change | 121 (50.4) | 85 (36.2) | 84 (34.1) |
|  | Minimally worse | 19 (7.9) | 12 (5.1) | 16 (6.5) |
|  | Much worse | 0 (0.0) | 3 (1.3) | 2 (0.8) |
|  | Very much worse | 0 (0.0) | 1 (0.4) | 0 (0.0) |

Data presented as mean±standard deviation or number of patients (%).

UPDRS=Unified Parkinson’s Disease Rating Scale; CGI-I=Clinical Global Impressions Improvement of illness.
