# Supplementary figures and images for: A Pooled Analysis From Phase 2b and 3 Studies in Japan of Istradefylline in Parkinson's Disease
Source: Mov Disord. 2020 Jun 5;35(8):1481–7. doi: 10.1002/mds.28095 (PMC7496465; doi:10.1002/mds.28095)

**Supplementary Figure 1.**

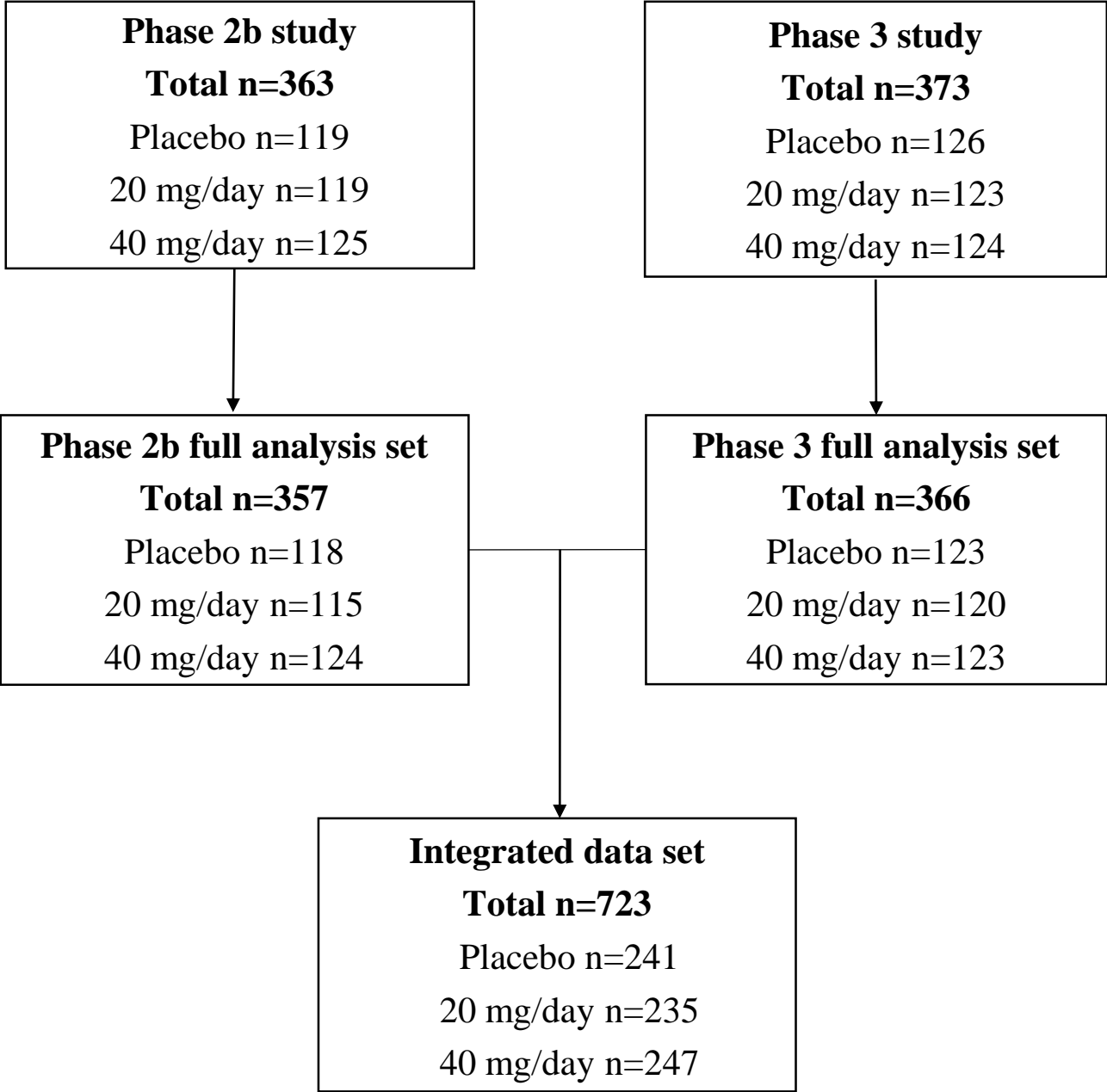

Supplementary Figure 2.

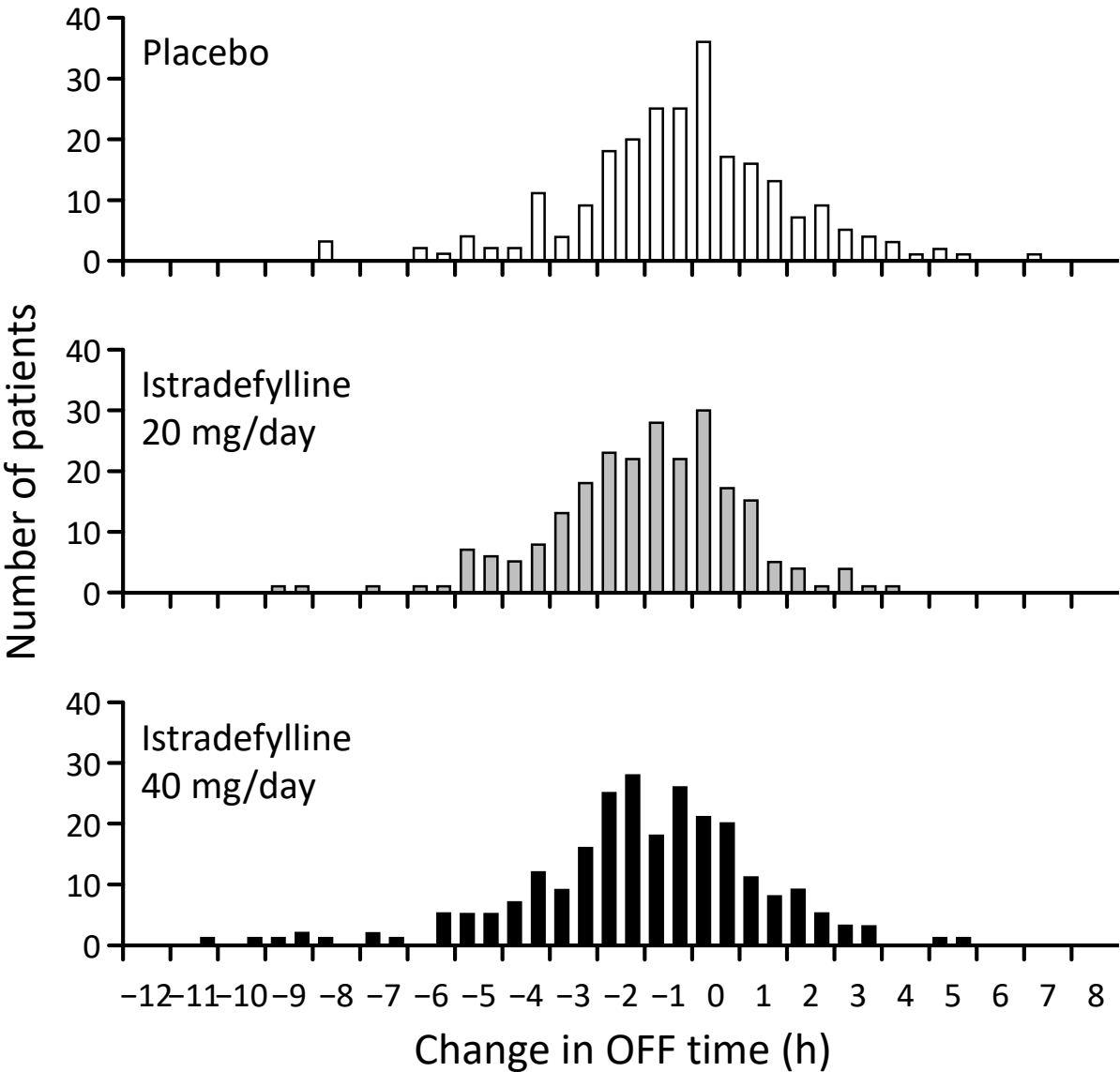

Supplement: Supplementary file 2 — Supplementary Figure 1 Patient disposition Supplementary Figure 2. Histogram showing the change from baseline at week 12 in daily OFF time in each treatment group Data are presented as means and standard deviations for each treatment group with p‐values for istradefylline versus placebo. [file MDS-35-1481-s002.pdf]
